# Buparlisib is a brain penetrable pan-PI3K inhibitor

**DOI:** 10.1038/s41598-018-29062-w

**Published:** 2018-07-17

**Authors:** Mark C. de Gooijer, Ping Zhang, Levi C. M. Buil, Ceren H. Çitirikkaya, Nishita Thota, Jos H. Beijnen, Olaf van Tellingen

**Affiliations:** 1 Division of Pharmacology, The Netherlands Cancer Institute, Plesmanlaan 121, 1066 CX Amsterdam, The Netherlands; 2 Mouse Cancer Clinic, The Netherlands Cancer Institute, Plesmanlaan 121, 1066 CX Amsterdam, The Netherlands; 3 Department of Neurosurgery, Qilu Hospital, Shandong University, Wenhua Xi Road 107, 250012 Jinan, P.R. China; 4 Department of Pharmacy and Pharmacology, The Netherlands Cancer Institute / MC Slotervaart Hospital, Louwesweg 6, 1066 EC Amsterdam, The Netherlands; 5 Division of Pharmacoepidemiology and Clinical Pharmacology, Department of Pharmaceutical Sciences, Faculty of Science, Utrecht University, Universiteitsweg 99, 3584 CG Utrecht, The Netherlands

## Abstract

Characterization of the genomic landscapes of intracranial tumours has revealed a clear role for the PI3K-AKT-mTOR pathway in tumorigenesis and tumour maintenance of these malignancies, making phosphatidylinositol 3-kinase (PI3K) inhibition a promising therapeutic strategy for these tumours. Buparlisib is a novel pan-PI3K inhibitor that is currently in clinical development for various cancers, including primary and secondary brain tumours. Importantly however, earlier studies have revealed that sufficient brain penetration is a prerequisite for antitumor efficacy against intracranial tumours. We therefore investigated the brain penetration of buparlisib using a comprehensive set of *in vitro* and *in vivo* mouse models. We demonstrate that buparlisib has an excellent brain penetration that is unaffected by efflux transporters at the blood-brain barrier, complete oral bioavailability and efficient intracranial target inhibition at clinically achievable plasma concentrations. Together, these characteristics make buparlisib the ideal candidate for intracranially-targeted therapeutic strategies that involve PI3K inhibition.

## Introduction

Phosphatidylinositol 3-kinase (PI3K) is a key component of the PI3K-AKT-mTOR pathway and as such important for cell proliferation and survival^1^. The pivotal role of PI3K in this signalling makes it an attractive anticancer target, especially in tumours harbouring an overactivated PI3K pathway^2^. Overactivation of the PI3K pathway is seen in many cancers, including primary intracranial cancers such as glioblastoma (GBM)^3^, diffuse intrinsic pontine glioma^4^ and paediatric high-grade glioma^4^, and cancers that frequently metastasize to the brain such as cutaneous melanoma^5^ and breast cancer^6^. PI3K inhibition has thus been proposed as a promising treatment strategy for various intracranial tumours^7–9^.

We and others have previously demonstrated that modest efficacy of PI3K inhibition can be achieved in preclinical mouse models of glioblastoma and brain metastases, but only if the compound used to inhibit PI3K exhibits sufficient brain penetration^10–14^. Therefore, it is important to assess whether a PI3K inhibitor has sufficient brain penetration prior to starting its development for treatment of intracranial cancers.

The brain penetration of a small molecular compound is generally restricted by the blood-brain barrier (BBB), which is composed of the brain endothelial cells (BECs) that are being supported by astrocytes and pericytes^15^. BECs abundantly express ATP-binding cassette (ABC) efflux transporters on their apical membranes that very efficiently pump xenobiotics back into the bloodstream, thereby protecting the brain parenchyma from potentially harmful substances. Among these transporters, P-glycoprotein (P-gp) and breast cancer resistance protein (BCRP) are the most dominant. Together, these efflux transporters are responsible for restricting the brain penetration of many anticancer drugs, be it classical chemotherapeutics or novel targeted agents^16^. In line with this, almost all PI3K inhibitors that have been analysed to date were demonstrated to be transported, ZSTK474 and GNE-317 appearing to be exceptions^12,13^.

Buparlisib is a novel pan-PI3K inhibitor that has been developed to inhibit all class I PI3K isoforms^17^. It has shown preclinical efficacy in various PI3K pathway overactivated cancer models, including GBM^18–20^. Buparlisib has thus far proceeded through phase I and phase II clinical trials in various extracranial solid tumours^21,22^ and is now also being tested in primary and secondary intracranial cancers (*e.g*., ClinicalTrials.gov Identifiers NCT02000882, NCT02452294, NCT01339052).

Despite some reports mentioning that buparlisib is BBB penetrable^17,18,21,23^, there are no pharmacokinetic data in the public domain supporting this claim. We here report a detailed analysis of the BBB penetration and oral bioavailability of buparlisib and demonstrate that it is a blood-brain barrier penetrable PI3K inhibitor with excellent oral bioavailability and intracranial target engagement.

## Results

### Buparlisib is only very weakly transported by murine BCRP *in vitro*

**Figure 1:**
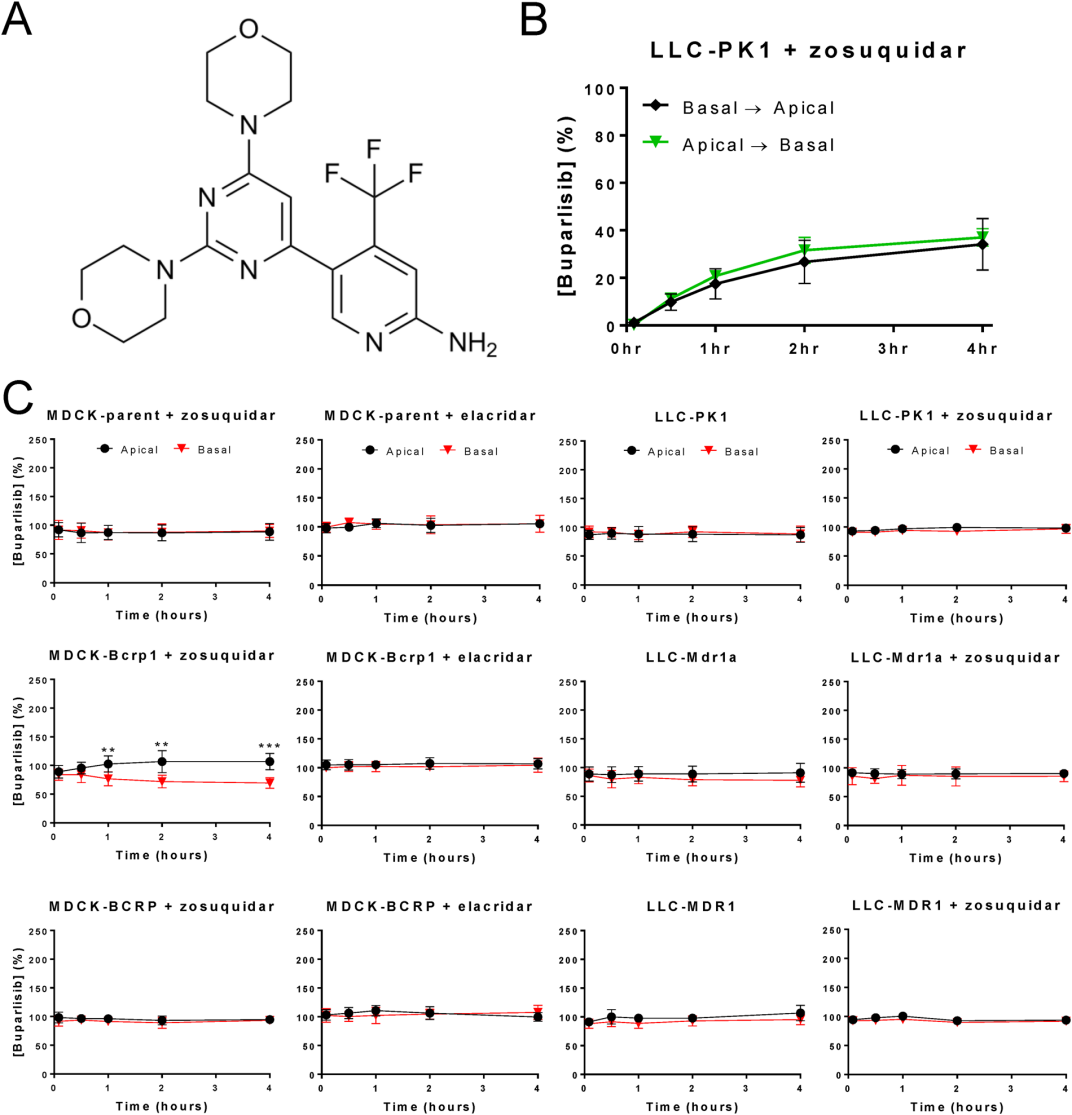
Analysis of buparlisib substrate affinity for ABC transporters using *in vitro* transport assays. (**A**) The chemical structure of buparlisib. (**B**) A conventional transport assay (CTA) using LLC-PK1 cells in presence of zosuquidar to block endogenous (porcine) P-gp activity. Buparlisib efficiently diffuses over a cellular monolayer irrespective of direction, plateauing to near-equilibrium in 4 hours. (**C**) Concentration equilibrium transport assays (CETAs) using MDCK or LLC cells that overexpress murine BCRP, (Bcrp1), human BCRP, murine P-gp (Mdr1a) or human P-gp (MDR1). No substrate affinity of buparlisib for BCRP, Mdr1a or MDR1 could be observed, whereas very minimal buparlisib transport was found in the MDCK-Bcrp1 cell line. The P-gp inhibitor zosuquidar was used in all MDCK cell lines to inhibit endogenous P-gp activity and in LLC cell lines to validate possible P-gp-mediated translocations. The dual BCRP/P-gp inhibitor elacridar was used to confirm possible BCRP-mediated translocations by abolishing buparlisib translocation in presence of elacridar. Data are represented as mean ± SD (n ≥ 4); **p < 0.01; ***p < 0.001.

We first sought to investigate whether buparlisib (Fig. 1A) is transported by P-gp or BCRP *in vitro* using concentration equilibrium transport assays (CETAs). CETAs are sensitive assays to determine translocation over a cellular monolayer and are frequently used to determine substrate affinity for ABC transporters by comparing cell lines that overexpress ABC transporters with their parental counterparts^24–26^. Since P-gp and BCRP are apically located transporters, a prerequisite for detecting substrate affinity for these transporters in a CETA is the capacity of a molecule to penetrate cell membranes. We therefore first confirmed that buparlisib could efficiently diffuse over a cellular monolayer using a conventional transwell set-up. In this set-up, buparlisib plateaued to almost complete equilibrium between the apical and basal compartments within 4 hours, independent of the direction of diffusion (Fig. 1B). This diffusion was studied in the parental LLC-PK1 cell line and in presence of the P-gp inhibitor zosuquidar to avoid any confounding effects of porcine P-gp on buparlisib diffusion.

Next, we studied buparlisib translocation in CETAs using cell lines that overexpress murine P-gp (Abcb1a/Mdr1a), murine BCRP (Abcg2/Bcrp1) or their human orthologues (ABCB1/MDR1, ABCG2). Only a minimal buparlisib translocation was observed in the MDCK-Bcrp1 CETA, whereas no translocation was found in any of the other CETAs, indicating that buparlisib is not a substrate of human P-gp, murine P-gp and human BCRP and a very weak substrate of murine BCRP (Fig. 1C). Note that functionality of the cell lines was confirmed as these assays were also performed in parallel using other compounds that did show basolateral to apical translocation.

### Buparlisib brain penetration is not restricted by P-gp and BCRP *in vivo*

**Figure 2:**
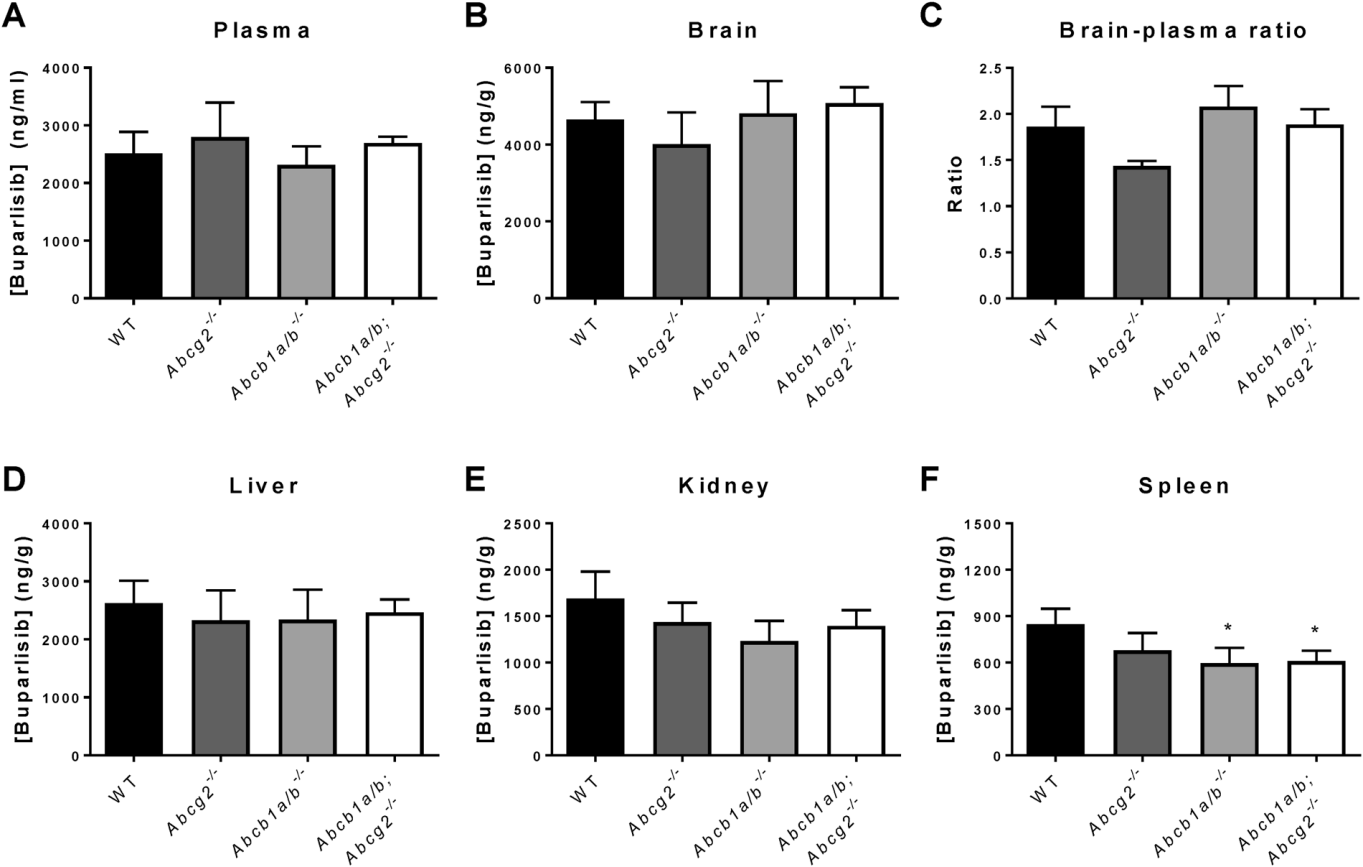
The impact of P-gp and BCRP on the brain and tissue penetration of buparlisib. Buparlisib was administered intravenously to wildtype, *Abcg2*^−/−^, *Abcb1a/b*^−/−^ and *Abcb1a/b*;*Abcg2*^−/−^ mice at a dose of 2 mg/kg. One hour after injection, blood and tissues were collected for LC-MS/MS analysis. No difference in buparlisib (**A**) plasma concentration, (**B**) brain concentration, (**C**) brain-plasma ratio, (**D**) liver concentration, (**E**) kidney concentration and (**F**) spleen concentration could be observed among the different mouse strains. All data are represented as mean ± SD (n = 4).

To study whether P-gp and BCRP attenuate buparlisib brain penetration *in vivo*, we measured the brain and plasma concentration in wildtype (WT), *Abcg2*^−/−^, *Abcb1a/b*^−/−^ and *Abcb1a/b*; *Abcg2*^−/−^ mice 1 hour after i.v. administration of 2 mg/kg buparlisib. No differences could be observed among the mouse strains in both the plasma and brain concentrations of buparlisib (Fig. 2A,B). These results demonstrate that P-gp and BCRP do not restrict the brain penetration of buparlisib *in vivo*. Interestingly, the brain-plasma ratio was between 1.5 and 2 in all strains, indicating that buparlisib exhibits excellent brain penetration (Fig. 2C). Remarkably, the brain concentration of buparlisib was even slightly higher than the concentrations in other well-perfused organs that contain fenestrated endothelium such as the liver, kidney and spleen (Fig. 2D–F).

### Buparlisib exhibits excellent oral bioavailability and achieves intracranial target inhibition

Buparlisib is given orally in both preclinical^19^ and clinical studies^21^, since it is claimed to have excellent oral bioavailability^17,18,21^. However, no data supporting this claim are available in the public domain. We therefore set out to determine the systemic exposure and brain concentrations after oral administration of buparlisib in mice.

**Figure 3:**
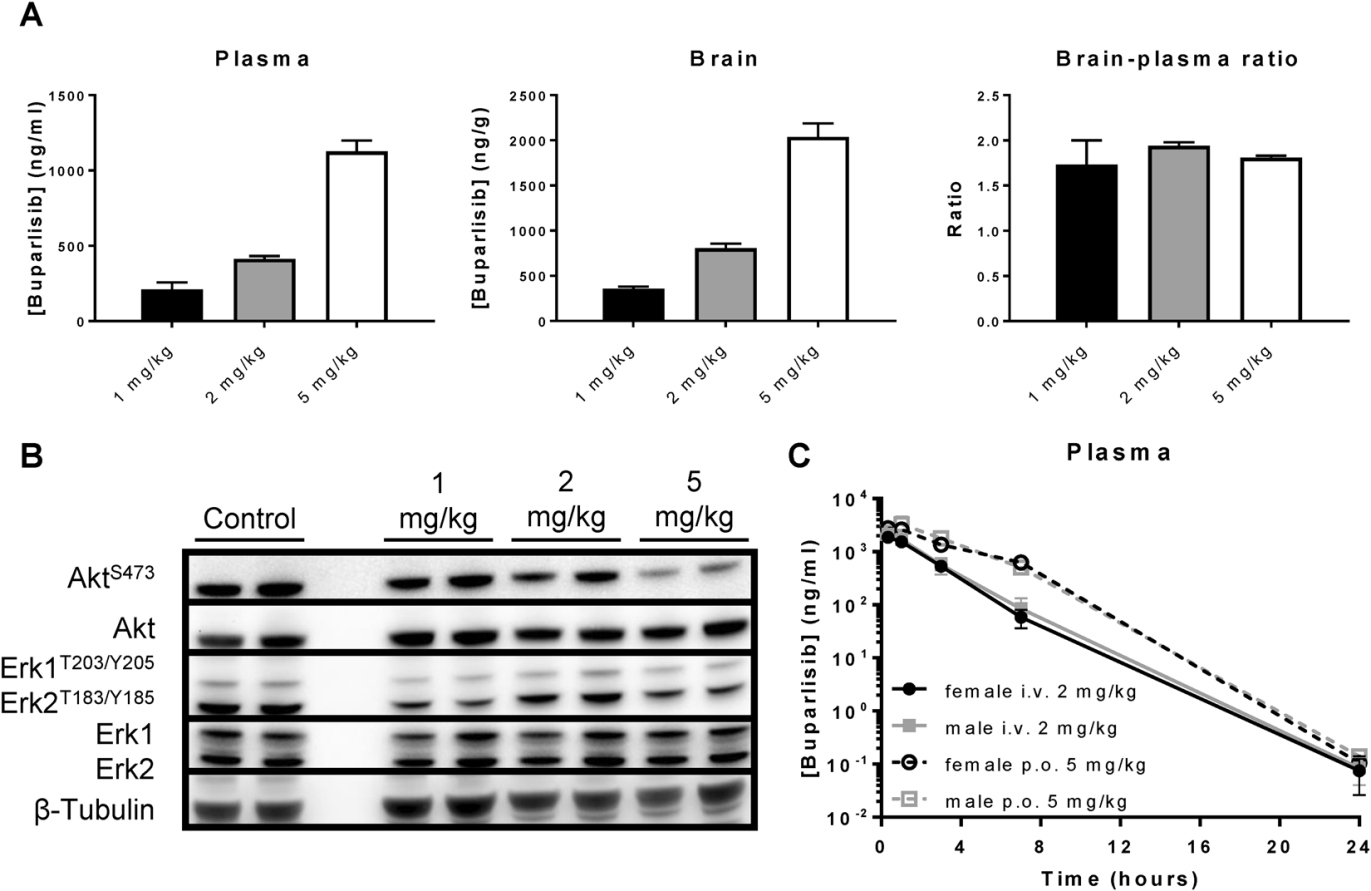
Buparlisib has excellent intracranial target engagement and oral bioavailability. (**A**) Buparlisib was orally administered to wildtype mice at a dose of 1 mg/kg, 2 mg/kg or 5 mg/kg. One hour after injection, blood and tissues were collected for LC-MS/MS analysis. Buparlisib plasma and brain levels increase dose-dependently, yielding similar and excellent brain-plasma ratios at all dose levels that were tested. (**B**) Immunoblotting of brain tissue lysates from (**A**). Buparlisib efficiently inhibited phosphorylation of Akt^S473^ in the brains of wildtype mice following oral administration at a dose of 5 mg/kg, without affecting signalling through ERK. (**C**) Plasma-time curves of male and female wildtype mice following intravenous (2 mg/kg) or oral (5 mg/kg) administration. No differences could be observed in buparlisib plasma pharmacokinetics among both genders. The oral bioavailability of buparlisib is excellent, since oral and intravenous administration yielded similar dose-adjusted AUCs (see Table 1). All data are represented as mean ± SD (n = 4).

First, we could confirm that buparlisib also achieves excellent brain penetration after oral administration, since the observed brain-plasma ratio in WT FVB mice was in line with those obtained following i.v. administration (Figs 2C, 3A). Interestingly, a dose-proportional increase in plasma and brain concentrations was observed, yielding similar brain-plasma ratios at three different dose levels. Although we only measured one time point, these data suggest that buparlisib displays linear pharmacokinetics between the 1 mg/kg and 5 mg/kg dose levels in mice.

Next, we studied the intracranial pharmacodynamics of buparlisib in WT FVB mice as measured by target inhibition. Immunoblotting of brain lysates revealed that buparlisib dose-dependently decreased the phosphorylation of AKT, the main target of PI3K, without affecting signalling through ERK (Fig. 3B). Importantly, efficient intracranial target inhibition could be reached in mice at clinically relevant plasma concentrations, since 5 mg/kg oral buparlisib resulted in plasma levels of approximately 1200 ng/mL (Fig. 3A)^21^.

Finally, we studied the pharmacokinetics of buparlisib in male and female mice and assessed the oral bioavailability in both genders by comparing the area-under-the-plasma concentration-time-curves (AUCs) following intravenous and oral administration. Both after oral and i.v. administration there was no difference in pharmacokinetics between male and female mice (Fig. 3C, Table 1). Strikingly, buparlisib displayed complete oral bioavailability since no difference in dose-corrected AUC could be observed between i.v. and oral administration in both genders. Interestingly, buparlisib exhibits a relatively small volume of distribution being less than 1 L/kg (Table 1), which is unusual for a small-molecule kinase inhibitor^27^. We therefore also assessed the buparlisib concentrations in a range of other tissues and indeed found that the levels were equal (liver) or even lower (kidney, spleen) than concurrent plasma levels (Fig. 2D–F). Thus, the brain contains the highest concentration of all tissues included in this study, suggesting that buparlisib may be a very suitable PI3K inhibitor candidate for treatment of intracranial tumours.

**Table 1:** Pharmacokinetic parameters of buparlisib after oral and i.v. administration to male and female FVB mice.

Administration route	Parameter	Time (h)	Gender	
			female	male
i.v. (2 mg/kg)	Plasma AUC (ng/ml^.^h)	0-∞	5600 ± 860	6300 ± 1500
	*C*_max_ (ng/ml)		1900 ± 410	2100 ± 190
	*t*_1/2_ (h)		1.60 ± 0.07	1.67 ± 0.07
	*V*_z_ (L/kg)		0.86 ± 0.14	0.80 ± 0.23
	*CL* (L/kg^.^h)		0.37 ± 0.06	0.33 ± 0.09
p.o. (5 mg/kg)	Plasma AUC (ng/ml^.^h)	0-∞	16000 ± 1700	16000 ± 1200
	*C*_max_ (ng/ml)		3000 ± 490	3300 ± 1300
	*t*_max_ (h)		0.67 ± 0.39	1.00 ± 0.00
	*t*_1/2_ (h)		1.40 ± 0.04	1.44 ± 0.06
	*F* (%)		112 ± 21.1	103 ± 26.1
	*V*_z_/*F* (L/kg)		0.63 ± 0.08	0.64 ± 0.03
	*CL*/*F* (L/kg.h)		0.32 ± 0.04	0.31 ± 0.02

AUC, area under the curve; *C*_max_, maximum concentration in plasma; *t*_max_, time to reach maximum plasma concentration; *t*_1/2_, elimination half-life; *V*_z_, apparent volume of distribution; *CL*, apparent clearance, *F*, oral bioavailability; *V*_z_/*F*, apparent volume of distribution after oral administration; *CL*/*F*, apparent clearance after oral administration. Data are represented as mean ± SD (n = 4).

## Discussion

This study demonstrates that buparlisib has pharmacokinetic properties that make it an attractive candidate for treatment of intracranial malignancies in patients. The accumulation of buparlisib in the brain is higher than in most other organs and buparlisib inhibits PI3K in the brain at clinically achievable plasma concentrations.

Characterization of the genomic landscapes of intracranial tumours has revealed a clear role for the PI3K-AKT-mTOR pathway in tumorigenesis and tumour maintenance of these malignancies^3–6^. Further work also supports that PI3K pathway hyperactivated tumours are vulnerable to PI3K inhition^2,18–20^. Thus, targeting this pathway is expected to be a promising therapeutic strategy for these tumours. Importantly however, sufficient brain penetration of any therapeutic agent is a prerequisite for antitumor efficacy against intracranial tumours^10–14,28^.

The ability of a compound to access the brain is largely determined by two factors, namely the ability to diffuse over cellular membranes and the affinity for the drug efflux transporters P-gp and BCRP that are expressed at the BBB. Of all PI3K inhibitors that have thus far been reported, buparlisib shows by far the best brain penetration. The brain-plasma ratio ranges between 1.5 and 2 (Figs 2C, 3A), whereas the brain-plasma ratio of several other PI3K inhibitors that are considered brain penetrable (NVP-BEZ235, ZSTK474 and GNE-317) do not exceed 1^12,13^. Importantly, the buparlisib brain concentration is also higher than in most other tissues, which is in line with the relatively low distribution volume of this drug (<1 L/kg; Table 1)^27^. The reason why buparlisib distributes more to the brain than to other tissues is unclear, but could be related to more preferential protein binding in the brain or substrate affinity for uptake transporters that are present at the BBB. Regardless of the cause, this characteristic makes buparlisib a clear frontrunner among all PI3K inhibitors for development against intracranial tumours provided that the mouse mimics the human in this respect.

The plasma concentration-time profiles of buparlisib in mice and humans are very different. The peak plasma levels in humans receiving the maximum tolerated dose of buparlisib of 100 mg per day is about 1700 ng/ml (4.15 μM) and remains above 800 ng/ml (1.95 μM) for the remaining period of 24 h until the next dose^21^. By contrast, the peak plasma level in mice receiving 5 mg/kg is in the same range as in humans, but steeply declines to below 1 ng/ml (2.4 nM) after 24 h. Notably, a plasma concentration between 500 and 1000 nM was needed for inhibition of PI3K in the brain of WT mice (Fig. 3A). Single agent antitumor efficacy in preclinical models has already been shown, but only at 30 to 50 mg/kg dose levels^17,18^. The lack of efficacy at lower doses is likely due to the short duration of pharmacologically active plasma levels. However, high dose levels will cause high and non-clinically relevant plasma levels during the first hours and create the risk that some of the profound single agent antitumor activities observed in mice are in fact due to off-target effects^29^.

Thus far, only one PI3K inhibitor has received FDA approval. Idelalisib is a class I PI3Kδ inhibitor and has been approved as monotherapy for treatment of follicular lymphoma and small lymphocytic lymphoma^30^. Buparlisib inhibits all class I PI3K isoforms. Next to the canonical function of class I PI3Kα in regulating tumour cell proliferation and survival, roles for other class I PI3K isoforms in the immune system, including tumour-induced immune suppression, are emerging^31,32^. Thus, pan-PI3K inhibition may be advantageous for antitumor efficacy. Buparlisib is at an advanced stage of development, with several phase III trials underway in extracranial malignancies (*e.g*., NCT01633060, NCT02756247) and phase II trials ongoing for primary and secondary intracranial tumours (*e.g*., NCT02000882, NCT01339052).

There is a growing body of clinical and preclinical evidence that PI3K inhibitors used in combination with other drugs may even be more promising for broader antitumor efficacy^33^. For instance in orthotopic mouse models of glioblastoma, the efficacy of PI3K inhibition as monotherapy has previously been shown to be modest at best, even when sufficient brain penetration and intracranial target inhibition could be reached^12,13^. Several preclinical studies have already pinpointed interesting strategies for combination treatment involving buparlisib, for instance combination with a MEK inhibitor^34^, Bcl-2 inhibitor^35^ and CSF-1R inhibitor^36^ in GBM or a Smo antagonist in medulloblastoma^37^. The finding that pharmacologically relevant plasma levels of buparlisib are achieved in patients warrants further clinical investigation of such combination therapies.

In summary, buparlisib is a pan-PI3K inhibitor with excellent brain penetration, complete oral bioavailability and efficient intracranial target inhibition at clinically achievable plasma concentrations, making it the attractive candidate for intracranially-targeted therapeutic strategies involving PI3K inhibition.

## Methods

### Drugs

Buparlisib (NVP-BKM120) and AZD8055 were purchased from Selleck Chemicals (Houston, TX) and zosuquidar from Eli Lilly (Indianapolis, IN). Elacridar was generously made available by GlaxoSmithKline (Research Triangle Park, NC).

### Cell culture

All cell lines used in this study were previously generated in our institute and generously provided by Dr. A.H. Schinkel^38–40^. All cells were cultured in MEM supplemented with 10% FBS, 1% L-glutamine, 1% sodium pyruvate, 1% MEM vitamins, 1% non-essential amino acids and 1% penicillin/streptomycin (all from Life Technologies, Carlsbad, CA) under 37 °C and 5% CO_2_ conditions.

### Concentration equilibrium transport assays

Conventional bidirectional transport assays (CTAs) and concentration equilibrium transport assays (CETAs) were performed as described previously^25^. Buparlisib was used at a concentration of 100 nM and, when appropriate, specific transport was inhibited using the P-gp inhibitor zosuquidar (5 μM) or the dual P-gp/BCRP inhibitor elacridar (5 μM). Transwell leakiness was determined as Carboxyl-[^14^C]-inulin translocation exceeding 1.5% per hour and these wells were excluded from the analysis.

To prepare buparlisib transport assay samples for LC-MS/MS analysis, 10 μL medium samples were mixed with 30 μL of acetonitrile:formic acid (100:1 v/v). After centrifugation, the supernatant was 5-fold diluted in water and the buparlisib concentration was measured using an LC-MS/MS system as described below.

### Animals

All animal housing and studies were approved by the Animal Experimental Committee of the Netherlands Cancer Institute and conducted according to national law and institutional guidelines. Mice were housed at 20.9 °C on a 12 hour light/dark cycle with food and water *ad libitum*.

### Pharmacokinetic studies

The pharmacokinetics of buparlisib were analysed in WT, *Abcg2*^−/−^, *Abcb1a/b*^−/−^ and *Abcb1a/b*; *Abcg2*^−/−^ FVB mice. All knockout mice strains have been developed within our institute^41–43^. For i.v. administration, buparlisib was dissolved in DMSO and injected at a dose of 2 mg/kg. For oral administration, buparlisib was formulated in DMSO:Cremophor EL:water (1:1:8 v/v/v) and administered at 1, 2 or 5 mg/kg as indicated. Tail vein bleeding was used to collect blood at intermediate time points, whereas at the last time point blood was drawn by cardiac puncture and various tissue were collected. Plasma was obtained from whole blood by centrifugation (5 min, 5,000 rpm, 4 °C). After weighing, tissues were homogenized using a FastPrep®-24 (MP-Biomedicals, NY) in 1% (w/v) bovine serum albumin in water. Buparlisib was extracted from plasma and tissue homogenate by liquid-liquid extraction with ethyl acetate using AZD8055 as an internal standard.

### LC-MS/MS analysis

Buparlisib was measured in samples from *in vitro* transport assays and *in vivo* pharmacokinetic studies using an LC-MS/MS system comprised of an UltiMate 3000 LC Systems (Dionex, Sunnyvale, CA) and an API 4000 mass spectrometer (Sciex, Framingham, MA). Samples were run through a Securityguard C18 pre-column (Phenomenex, Utrecht, The Netherlands) prior to separation on a ZORBAX Extend-C18 column (Agilent, Santa Clara, CA). Elution was done using a in a 5 minute gradient from 20% to 95% B (mobile phase A was 0.1% HCOOH in water (v/v) and mobile phase B was methanol). 95% B was maintained for 3 min followed by re-equilibration at 20% B. Multiple reaction monitoring was performed at 411.3/367.2 (buparlisib) and 418.2/138.4 (AZD8055). Analyst^®^ 1.6.2 software (AB Sciex; Foster City, CA) was used for system control and data analysis.

### Western blotting

Mouse brains were homogenized in RIPA buffer supplemented with sodium fluoride (2 mM), sodium orthovanadate (1 mM), sodium pyrophosphate (1 mM), β-glycerophosphate (2.5 mM), PMSF (1 mM), DTT (1 mM) and protein inhibitor cocktail (Roche; Basel, Switzerland). The primary antibodies that were used in this study are phospho-Akt^S473^ (1:1000; D9E, #4060; Cell Signaling Technology; Danvers, MA), total Akt (1:1000; #9272; Cell Signaling Technology), phospho-Erk1^T202/204^/Erk2^T183/Y185^ (1:1000; sc-16982; Santa Cruz Biotechnology, Dallas, TX), total Erk1/2 (1:1000; C16, sc-93; Santa Cruz Biotechnology) and β–tubulin (1:1000; T3952; Sigma-Aldrich, St. Louis, MO). Goat-anti-rabbit-HRP (1:2000; DAKO, Santa Clara, CA) was used a secondary antibody.

### Pharmacokinetic and statistical analysis

As described in detail before, CETA results were analysed with the General linear model repeated measures procedure of SPSS (v22; SPSS Inc, Chicago, IL)^25^. PK solver was used to determine pharmacokinetic parameters^44^. The standard error of the oral bioavailability was calculated using the formula below:

$$S{E}_{F}=F\sqrt{{(\frac{S{E}_{AU{C}_{p.o.}}}{AU{C}_{p.o.}})}^{2}+{(\frac{S{E}_{AU{C}_{i.v.}}}{AU{C}_{i.v.}})}^{2}}$$

Buparlisib concentrations in WT and transporter knockout mice *in vivo* were compared using one-way analysis of variance followed by *post hoc* Bonferroni tests. Statistical differences between the buparlisib PK parameters in male and female mice were calculated as Bonferroni corrected *p*-values using multiple Student’s t-tests. Differences were considered statistically significant when *p* < 0.05.
